# Early Detection of Testicular Sertoli Cell Tumor Through Physical Examination: A Case Report

**DOI:** 10.7759/cureus.75891

**Published:** 2024-12-17

**Authors:** Yossy Machluf, Majd Said, Yigal Chechik, Eduardo Cohen, Yoram Chaiter

**Affiliations:** 1 Medical Corps, Israel Defense Forces, Rehovot, ISR; 2 Medical Corps, Israel Defense Forces, Haifa, ISR; 3 Medical Corps, Israel Defense Forces, Ramat Gan, ISR

**Keywords:** adolescents, germ cell tumors, sertoli tumor, testicular self-examination, testicular tumors

## Abstract

Testicular tumors represent the most common solid organ malignancy in young and adult males. Sex cord-stromal tumors are the second-largest group of primary testicular cancers, after germ-cell tumors. Sertoli cell (SC) tumors of the testis are relatively rare, accounting for only a small fraction of testicular tumors. Here, we report on a 17-year-old male diagnosed with a testicular tumor, which was identified during a routine physical examination at a military recruitment center. A hard lump was detected on the lower pole of the left testicle. Ultrasonography confirmed a solid lump with lobular borders. A left orchiectomy was performed, with pathology findings consistent with a SC tumor. Imaging showed no evidence of secondary tumor spread. Follow-up did not detect a recurrence of the tumor or any signs of spread. This case strengthens the importance of physical examination of male genitalia, particularly by experienced medical physicians, that may detect testicular tumors and prevent morbidity and mortality. This case study may add to the ongoing debate and inconsistent recommendations on the need for testicular examination in general and, more specifically, testicular self-examination or testicular examination by the physician.

## Introduction

Testicular tumors represent the most common solid organ malignancy in males aged 15-35 [1]. Sex cord-stromal tumors (SCST) are the second-largest group of primary testicular cancers (TCa) after germ cell tumors [2]. Of SCSTs, the rare Sertoli cell (SC) tumor of the testis accounts for only 1% of testicular tumors [3]. While the relative rate of metastatic disease at presentation among patients with SC tumors compared to those with germ cell tumors is still controversial [3,4], the cancer-specific mortality rates are higher in SC tumors compared to Leydig and germ cell tumors [4].

The radiographic features of SC tumors of the testis, as evident by ultrasound analysis, are ill-defined hypoechoic intratesticular lesions, which are usually solitary, unless part of a syndrome [1]. Moreover, CD99, vimentin, and α-inhibin are among the well-established immunohistochemical markers for identifying SC tumors and SCST [3,5], while these tumors are usually positive also for cytokeratin and S-100, though weakly. CD99 is a marker for MIC-2; it reacts with normal Sertoli cells and granulosa cells, and its degree of reactivity correlates with the degree of differentiation in Sertoli-Leydig cell tumors. Vimentin is an intermediate filament protein that primarily provides mechanical support, preserves cell shape, and maintains the nuclear position, and it is often used as a marker to identify Sertoli cells. Inhibin, a gonadal protein that belongs to the transforming growth factor (TGF) beta superfamily and plays a role in regulating the secretion of pituitary follicle-stimulating hormone through a negative feedback mechanism, is produced by normal testicular stromal cells (TSC). It is expressed and produced in the majority of human TSC tumors, and it is a useful marker for human TSC tumors with intense immunohistochemical staining of inhibin-α occurring typically in >90% of Leydig and SC tumors but in only about 10% of testicular germ cell tumors [2,5].

Testicular self-examination (TSE) can facilitate early detection and identification of tumors and other testicular lesions, potentially influencing survival rates, quality of life, and the likelihood of future health complications [6]. Yet, while TSE is a well-established recommendation for patients with a history of TCa, its utility in healthy young men is less frequent and even unclear [7]. We present a rare case of a 17-year-old male with an SC tumor that was detected during the medical process at an Israel Defense Forces (IDF) recruitment center.

## Case presentation

The medical process at recruitment centers

The Israeli National Military Service Act obligates most citizens to serve in the IDF. As part of the draft process, all 16.5-year-old Israelis undergo comprehensive medical evaluation at regional recruitment centers [7]. This involves preliminary documentation from the primary care physician and diverse paramedical and medical tests. Examination at the recruitment center comprises a systematic anamnesis, psychological evaluation, complete physical examination, and referral for further investigation, if necessary.

Complete physical examination and anamnesis

In August 2019, a male 17-year-old potential IDF recruit (hereafter "patient") underwent a medical assessment. He reported migraines, occasional cigarette use, and a familial history of epilepsy but was otherwise healthy. The findings of the paramedical tests (urinalysis, blood pressure, and pulse) and measurements (height and weight) were all within the normal ranges. A vision test revealed myopia in both eyes. An initial examination noted genu varum leg deformity.

Genital findings and further imaging

**Figure 1:**
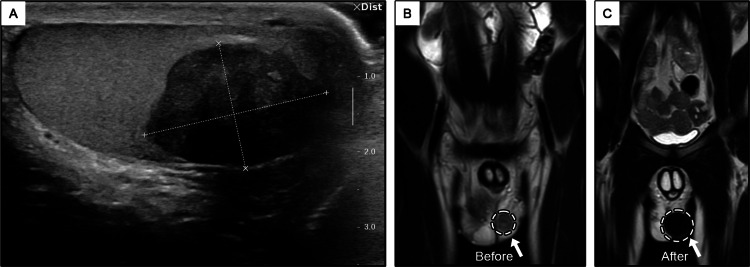
Imaging of the left testicle: (A) Ultrasound (US) imaging illustrating the size of the tumor. (B) Magnetic resonance imaging (MRI) showing the tumor in the left testicle (before surgery) and (C) replacement by prosthesis (after surgery).

During the genital examination, the examining physician detected a hard lump on the lower pole of the left testicle. The patient had no prior knowledge of any genital issues; therefore, he was referred for further tests. He underwent an ambulatory testicular ultrasonography (US) in October 2019, which demonstrated a normal-looking right testicle, whereas the left testicle demonstrated a hypoechoic tissue structure with regular borders up to 2.2 cm in size with weak internal blood flow (Figure 1A). These features, namely hypoechoic structure and lobulated margins, are in accordance with SC tumor diagnostic criteria. The head of the epididymis and the epididymis were not thickened. The pampiniform plexus was not dilated. There was no excess fluid around the testicle. The patient was referred to a urologist, who decided to perform another US, including Doppler, to explore a possible space-occupying lesion in the left testicle. The US, which was performed in late October, showed a right testicle of normal size and texture and a cystic finding with thick contents measuring 3.8 × 6.5 mm in the subcutaneous tissue of the right scrotum. The left testicle was of normal size; in the lower septum, a non-echoic, uneven solid lump was demonstrated, with lobular borders, measuring 17 × 25 mm. The appearance of the finding was suspicious, with no evidence of fluids around the testicles. The Doppler examination demonstrated normal, equal blood flow in both testicles. The epididymis was normal on both sides. To conclude, a space-occupying lesion was confirmed in the left testicle. Blood tests were taken for the markers alpha-fetoprotein, beta human chorionic gonadotropin, and lactate dehydrogenase, which were within the normal ranges. A systemic investigation was performed that included a chest computed tomography (CT) scan, which showed no suspicion of secondary spread to the lungs, and abdominal pelvic magnetic resonance imaging (MRI), which showed neither lymphadenopathy nor evidence of secondary spread.

Referral to radical orchiectomy, pathological findings, and monitoring

The patient was admitted for radical orchiectomy on the left testicle in late October 2019, in the urology department at a hospital in northern Israel. At his request, the radical orchiectomy on the left side included the placement of a prosthesis (Figure 1B vs. Figure 1C).

**Figure 2:**
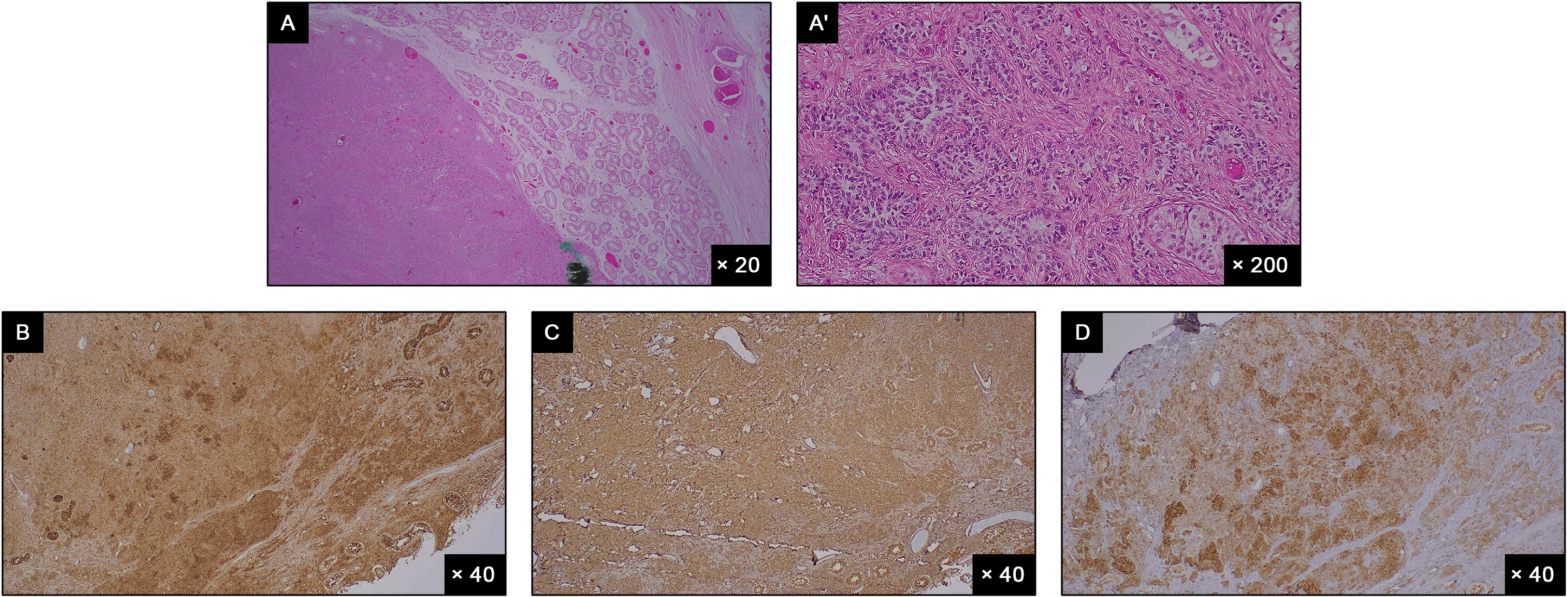
Staining sections of the tumor tissue for different markers: (A) hematoxylin and eosin staining; immunohistochemical staining for sex cord stromal tumor markers (B) CD99, (C) vimentin, and (D) α-inhibin.

The pathology results showed a sex cord-gonadal stromal tumor, consistent with SC tumor (Figure 2), with a maximum size of 1.7 cm. It was immunohistochemically positive for CD99, vimentin, α-inhibin, calretinin, and Melan-A (focally weakly; data not shown). There was no infiltration into the tunica albuginea, epididymis, or rete testis; the spermatic cord, including resection margins, was free of tumor; no lymphovascular invasion was observed; Leydig cells were within normal limits; and there was no observation of marked nuclear atypia or pleomorphism, increased mitosis or necrosis, or extra-testicular extension. The tumor was classified as STAGE IA, with T1A, N0, and M0.

Almost a month after the surgery, a follow-up by a urologist was normal, including a prosthesis in place without suspicion of postoperative infection. In a follow-up after three and a half months, the patient felt well without any complaints. It was decided that the patient was not a candidate for chemotherapy treatment; rather, an annual imaging follow-up was recommended if the first imaging after the operation was normal. In March 2020, abdominal and pelvic CT imaging was performed, showing no evidence of recurrence, and markers were also normal. Re-examination almost a year later by a urologist found no evidence of recurrence: all the follow-up and tests (MRI of the abdomen and pelvis, chest X-ray, markers) were normal. According to the Israeli National Military Service Act, the patient was exempt from service, yet he decided to enlist as a volunteer.

## Discussion

SC and Sertoli-form tumors of the testis are rare, thereby posing challenges for their differential diagnosis and classification [8]. Currently, the importance and efficacy of conducting physical examinations of the male genitalia are under ongoing debate, even in the cases of highly malignant tumors that bear a high risk of rapid progression to metastatic disease. The health benefits of screening have been questioned due to the low incidence of TCa and favorable outcomes of effective treatment procedures even in advanced stages, which lead to a very high survival rate. Thus, the U.S. Preventive Services Task Force published a Grade D recommendation for both TSE and clinical evaluation to screen for TCa in asymptomatic males [9]. Also, other pivotal organizations, like the National Cancer Institute, the National Comprehensive Cancer Network, and the American Academy of Family Physicians, do not recommend TSE. Whereas a previous literature review showed no evidence against this recommendation [10], recent emerging evidence has demonstrated the benefits of TSE [5] and requested a rating adjustment to grade B for both the clinical setting and self-examination [11].

In contrast, some advocate for interventions to increase men's awareness of TCa and TSE, which may include a PowerPoint presentation, an online educational brochure, high-quality video-assisted tools, a motivational video, and a virtual reality game that provides reliable, comprehensive information and instructions for performing TSE, which may contribute to both men's greater knowledge and more extensive and effective performance of TSE [12-17]. The European Association of Urology and American Urology Association advocate TSE in their guidelines, yet it is recommended to men in high-risk groups, which include a history of cryptorchidism, as well as those with a personal or family history of TC [18]. This limited recommendation to a specific at-risk population may be due to concerns of false-positive non-cancerous findings (much higher prevalence of benign scrotal conditions and other testicular lesions), which can increase unnecessary referrals and costs and raise anxiety. However, Rovito and colleagues concluded that TSE is a behavior that is beneficial beyond detecting cancer, with clear “off-label” contributions to promote testicular health, self-awareness, and wellness among males [19]. In addition, a recent review highlighted the importance of promoting "innovative educational interventions aimed at younger men, whilst raising their awareness of testicular disorders and increasing their help-seeking intentions for testicular disorder symptoms" and encouraging clinicians "to instruct men to familiarize themselves with the look and feel of their own testes and to seek timely medical attention for abnormalities" [13].

Nevertheless, one should bear in mind other potential caveats of TSE: (I) TSE faces stereotypes and demographic and cultural barriers in different populations (ethnicities, orthodox religious groups, etc.); (II) TSE also poses various challenges in terms of the required skills. These can be overcome by a periodic or routine testicular examination and educative instruction on TSE by physicians. The proficiency of physicians who conduct thousands of such examinations daily is noteworthy. At least in military settings, there is a pressing need for comprehensive medical education on TCa [20].

Our case report, with its related characteristics of the medical profiling process at military recruitment centers, further supports and strengthens the key role of screening for early detection of SC tumors. The medical physician is pivotal in the screening process and may also contribute to future effective TSE. Furthermore, our long-standing experience advocates for male genitalia examination by physicians, as performed regularly at recruitment centers in Israel, which may detect not only testicular tumors but also other testicular lesions - such as varicoceles, hydroceles, hernias, funiculoceles, cysts, and cryptorchidism - that affect subjects' health and quality of life.

## Conclusions

Physical examination of male genitalia is performed regularly at recruitment centers in Israel. It may detect varicoceles, hydroceles, hernias, funiculoceles, cysts, cryptorchidism, and, rarely, testicular tumors. Early detection of tumors by physical examination allows removal by orchiectomy and cure. Periodic physical examination, either self-administered or in clinical settings, can prevent significant future morbidity and even mortality caused by such malignant tumors.
